# A case of sigmoid volvulus in an unexpected demographic

**DOI:** 10.1186/s40792-020-01105-3

**Published:** 2021-01-11

**Authors:** Mohammad Saba, Joshua Rosenberg, Gregory Wu, Gudata Hinika

**Affiliations:** 1 Ross University School of Medicine, 2300 SW 145th Ave, Suite 200, Miramar, FL 33027 USA; 2 Department of Surgery, California Hospital Medical Center, 1401 S Grand Ave, Los Angeles, CA 90015 USA

**Keywords:** Sigmoidectomy, Volvulus, Bowel obstruction, Emergent surgery, Sigmoid colon

## Abstract

**Background:**

A sigmoid volvulus occurs when a segment of the colon twists upon its mesentery. This infliction is associated with old age, multiple co-morbidities, and the male sex. We present a rare case of sigmoid volvulus that occurred in a healthy young female.

**Case presentation:**

A 28-year-old female presented with a one week history of constipation and abdominal pain. Her symptoms suddenly worsened and became associated with vomiting and severe pain. A focused history taking and physical examination showed peritoneal signs that led to timely diagnostic imaging to be implemented. Computed tomography (CT) of the abdomen was consistent with sigmoid volvulus. Our patient underwent emergent laparotomy with a sigmoidectomy and recovered with no post-operative complications.

**Conclusion:**

This case report emphasizes the importance of clinicians maintaining a sigmoid volvulus as a rare, yet important differential when approaching abdominal pain in young healthy patients.

## Background

Colonic volvulus accounts for around 2% of all bowel obstructions in the United States [1]. This number can approach 50% in many other parts of the world, where colonic volvulus is considered an endemic [1]. More specifically, the involvement of the sigmoid colon consists of the overwhelming majority of colonic volvulus cases, possibly 60.9–80% [2, 3].

Sigmoid volvulus has classically been described in patients in the setting of chronic constipation. Therefore, the elderly are typically affected by this form of large bowel obstruction. In addition to the elderly, institutionalized neuropsychiatric patients are also considered when evaluating the risk factors of a sigmoid volvulus [4].

Sigmoid volvulus can be a life-threatening emergency. The rotation of the colon on its mesentery can lead to complete obstruction of the bowel lumen. This may lead to severe signs and symptoms such as abdominal distention, vomiting, constipation, and fluid-electrolyte imbalances. A more detrimental complication that must be anticipated is a strangulation of the mesenteric vasculature leading to bowel ischemia and necrosis [5]. Given the direness of this debilitating affliction, prompt identification and management is critical.

## Case presentation

A 28-year-old female with no significant past medical history was apparently healthy until presenting to the emergency department with 1 week of worsening diffuse abdominal pain. Her intermittent pain had suddenly worsened over night which prompted her hospital visit. This sudden escalation in pain was also accompanied with multiple episodes of vomiting. She had been constipated with no bowel movement for the entire week prior to presenting to the hospital. The patient stated to have a well-balanced diet and live an active lifestyle for most of her life.

**Fig. 1:**
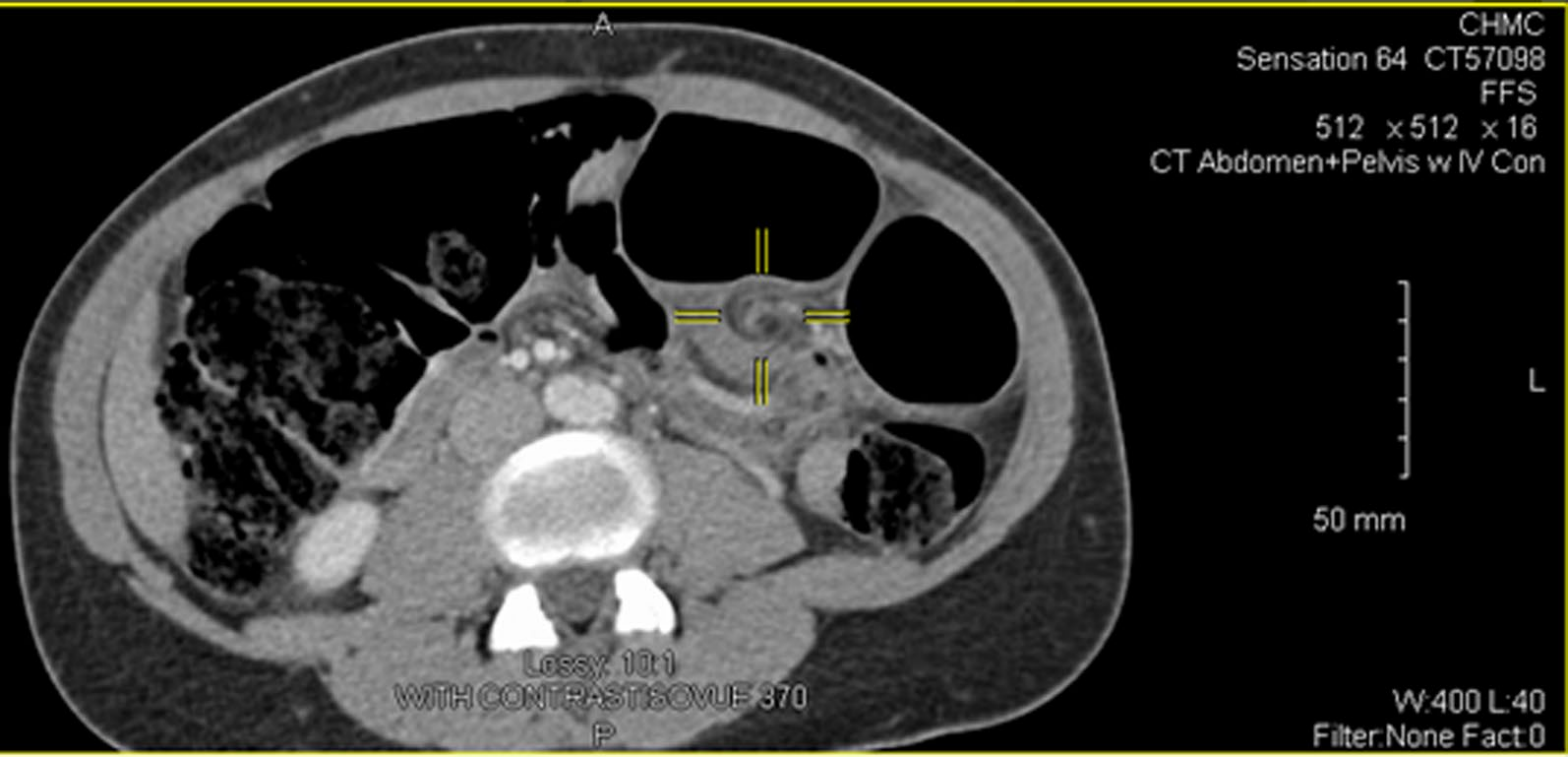
Axial CT showing “whirl” sign (emphasized by the yellow cursor) and distended large bowel

Upon physical examination, the patient was in serious distress due to pain. Her vital signs were within normal limits. Her abdomen was very tender to palpations in all four quadrants. Rebound tenderness was also present. These signs and symptoms were of major concern, as they pointed towards the patient having developed peritonitis. A computed tomography (CT) of the abdomen and pelvis was conducted and was highly suggestive of a sigmoid colon volvulus. The CT showed a largely dilated colon with a pathognomonic “whirl” pattern present (see Fig. 1).

**Fig. 2:**
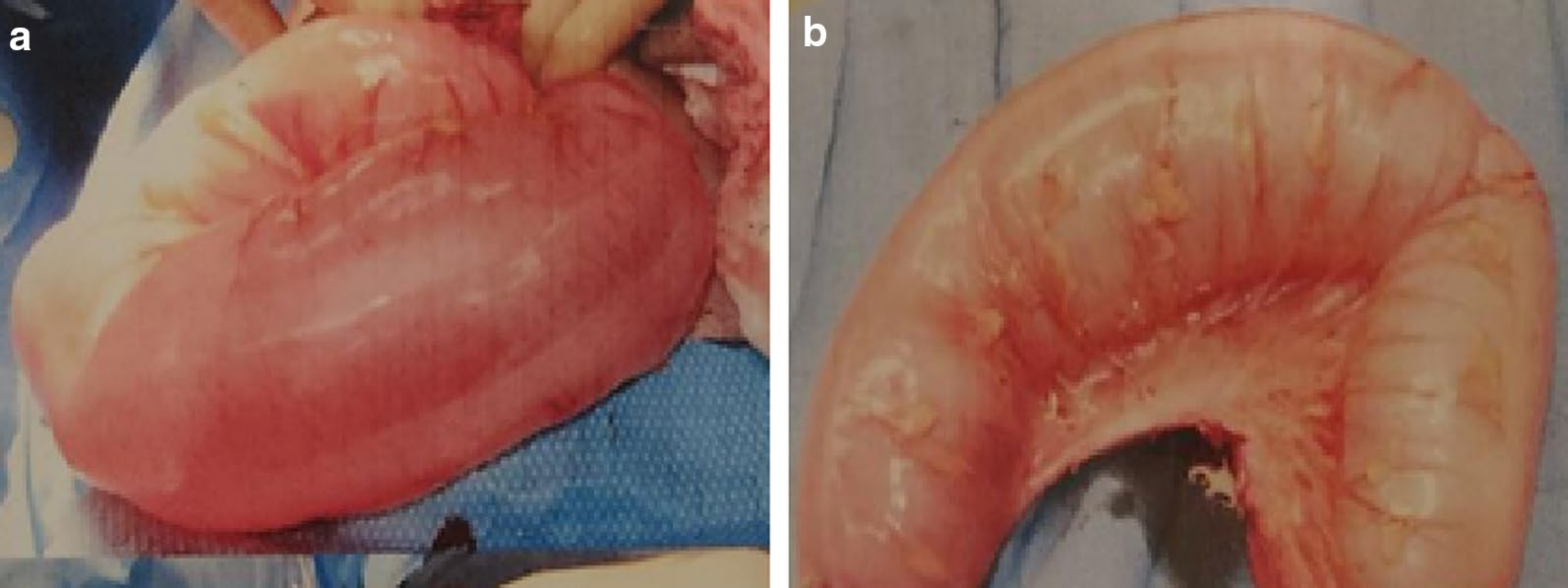
Intraoperative images of excised sigmoid colon. **a** Image taken from prior to resection. **b** Image taken after resection. Note the distended bowel which spans the entire width of the abdomen

Clinical suspicion for potential bowel necrosis was high given the patient’s peritoneal signs and sudden worsening of symptoms. Therefore, the patient underwent an emergent laparotomy. A sigmoidectomy of the distended portion of bowel was performed (see Fig. 2). The remaining proximal and distal ends were inspected to be clean and a primary anastomotic continuity of the bowel was created. Intra-operatively, we noted an abnormally redundant sigmoid colonic mesentery.

**Fig. 3:**
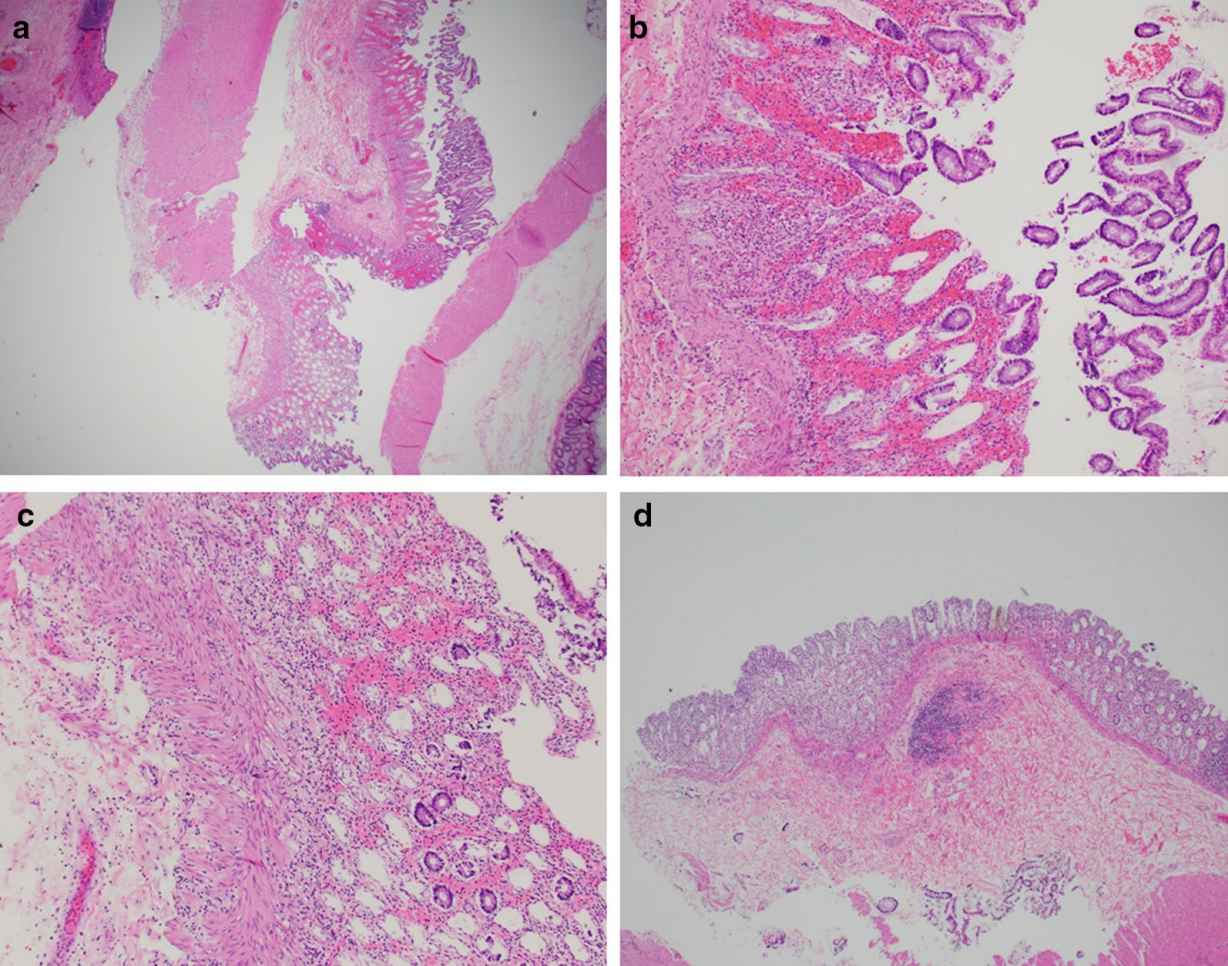
Histopathological analysis of the excised sigmoid colon showing ischemic changes. **a**–**c** Sigmoid colonic section showing mucosal sloughing, mucosal hemorrhages, and fibrin deposition which are all indications of ischemia; H&E stain 10 × (**a**), 2 × of upper portion of image **a** (**b**), 4 × of lower portion of image **a** (**c**). **d** Similar ischemic changes seen in a section from a different site of the excised tissue; H&E stain 10 ×

The pathology report of the resected specimen revealed necrotic bowel with evidence of ischemic changes and focal ulcerations. Ischemic changes seen on microscopy included mucosal sloughing, mucosal hemorrhages, and fibrin deposition (see Fig. 3). The patient recovered without complication with return to enteral nutrition and resumed bowel function. She was subsequently discharged on post operative day number five.

## Discussion

Certain important factors must be emphasized for our case to be of beneficial contribution to the current literature. First, the demographic of our patient is of interest. A vast epidemiological study conducted by Halabi et al., reviewed 19,220 sigmoid volvulus (SV) cases [1]. This large sample size showed that the mean age for the onset of SV was 70 years and was consistent with what is commonly known in the medical community. However, SV cases have been noted in healthy young adults as well [6, 9, 12, 13]. Our findings also contribute to the interesting outliers in the demographic for SV.

Common clinical presentations exist between young and elderly SV patients. These symptoms include, but are not limited to colicky pain, constipation, abdominal distention, nausea, and vomiting [1–25]. However, we noted a unique pattern in the scarce literature that addresses SV presentations in young adults such as our patient. This pattern is in regards to the duration of time from when symptoms first appear to the time at which patients present. We labeled this duration variable as Symptom to Presentation Time (SPT).

Our analysis of literature similar to this case report that expressed SV in rare young demographics (specifically in late adolescence and young adulthood) yielded a higher SPT than what has been reported for the elderly. Atamalp et al. showed that for 381 SV patients who were 60 years or older, the mean SPT was 42.5 h [14]. Welch and Anderson showed that 72% of their SV patients who had a mean age of 72 years, presented with symptoms in less than 48 h duration [15]. In comparison with these large studies, our analysis of the few rare case reports and case series regarding younger patients, discovered that most SPT’s were more than four days and many up to a week (see Table 1) [6–13].

**Table 1:** Duration of symptoms to presentation in rare cases of young sigmoid volvulus cases

Authors	Age and sex of patient(s)	Duration of symptoms prior to presentation (SPT)
Cook and Allison (1984) [6]	An 18 year-old male	5 days
Krausz et al. (1979) [7]	a. A 20 year-old (sex not specified)	a. 7 days
	b. A 19 year-old female	b. 5 days
Krupsky et al. (1987) [8]	A 17 year-old female	7 days
Sarfaraz et al. (2017) [9]	a. A 25 year-old male	a. 7 days
	b. A 35 year-old male	b. 3 days
	c. A 25 year old male	c. 3 days
	d. A 25 year-old male	d. 2 days
Slidell et al. (2014) [10]	An 18 year-old male	7 days
Udezue (1990) [11]	60 total patients, 18–40 years-old; 55/60 were below 30 years-old; (sex varied)	75% of the patients presented at ≥ 4 days, with some reported as long as 7 days
Ugur et al. (2014) [12]	A 17 year-old female	4 days
Weingrow et al. (2012) [13]	A 25 year-old female	5 days

We acknowledge that more data such as these rare reports are needed to come to any concrete conclusions about this discrepancy in SPT between young and old patients. However, this preliminary observation of prolonged SPT in younger adults further emphasizes the necessity of prompt diagnosis of SV in young patients. This is especially true when considering that clinicians may already have a low suspicion of SV due to rare incidence in younger individuals; as any delay in diagnosis in addition to an already delayed presentation could be catastrophic.

In addition to making expeditious diagnosis of SV, it is just as important to further identify if ischemia or gangrenous necrosis is present. This differentiation can determine an emergent surgical approach as compared to the sigmoidoscopic detorsion and delayed resection which continues to be the standard of care for non-emergent SV [16]. This case further shows that a colonic resection with a primary anastomosis of an unprepped bowel can be applied in an emergent SV without complications. Our approach is consistent with what has been reported in the literature [17–19].

It is worth noting that a laparoscopic approach to sigmoid volvulus has been documented in the literature [20, 21]. However, this method remains controversial in the emergent setting due to lack of evidence in the literature and the reliance on expert surgical skill [1, 20, 22]. Authors have also expressed the importance of emphasizing open laparotomy compared to the laparoscopic approach in relation to anastomotic failure, simplicity of technique, and recurrence rates [23]. We conducted a routine open laparotomy for this case.

Imaging studies are also essential to the diagnostic process for patients suspected of having sigmoid volvulus. An abdominal film may present air-fluid levels and dilated large bowel, with a possible bent appearance, also known as the “coffee bean” sign [1, 16]. A gastrografin or barium enema can show the tapering effect of the narrowed twisted lumen of the obstructed colon [5, 16]. Finally, as seen in our case, computed tomography can further detect the twisting pattern that occurs in volvulus in a phenomena dubbed the “whirl” pattern [24].

As a final note, it is important to discuss the possible etiologies for the rare occurrence of volvulus in a young healthy patient. Both acquired and congenital anatomical redundancies in the mesentery are possible etiologies. Acquired redundancy has been attributed to chronic constipation due to a high-fiber diet, sedentary lifestyle, and neurological disease [1, 18]. As mentioned before, these etiologies are commonly seen in the elderly. Congenital issues include being born with a redundant broad mesentery with a narrow base, malfixation of the mesentery, and Hirschsprung disease [1, 5, 25]. Given the young age, healthy past medical history of our patient, and mesenteric redundancy seen intra-operatively in our patient, we are inclined to believe her case was due to a congenital anatomical defect. Since screening for such an occurrence as seen in our patient is unfeasible, mindfulness of clinicians is critical to prompt diagnosis and management.

## Conclusion

This case report emphasizes the importance of clinicians maintaining a sigmoid volvulus as a rare yet dangerous differential when approaching abdominal pain in young healthy patients. We noted an interesting pattern regarding symptom-to-presentation duration in rare cases of SV in young patients found in the literature. We also demonstrate the emergent management of a sigmoid volvulus utilizing a resection with primary anastomosis of an unprepped bowel. Furthermore, we reiterate the importance of a classic imaging modality, the “whirl” sign, in helping establish diagnosis.

## Data Availability

Not applicable.

## Abbreviations

CT: Computed tomography
SV: Sigmoid volvulus
SPT: Symptom to presentation time
